# Formulation and Development of Transferrin Targeted Solid Lipid Nanoparticles for Breast Cancer Therapy

**DOI:** 10.3389/fphar.2020.614290

**Published:** 2020-11-27

**Authors:** Geeta S. Bhagwat, Rajani B. Athawale, Rajeev P. Gude, Shadab Md, Nabil A. Alhakamy, Usama A. Fahmy, Prashant Kesharwani

**Affiliations:** ^1^ H. K. College of Pharmacy, Mumbai, India; ^2^ Prin. K. M. Kundanani College of Pharmacy, Mumbai, India; ^3^ Advanced Centre for Treatment Research and Education in Cancer, Tata Memorial Centre, Navi Mumbai, India; ^4^ Department of Pharmaceutics, Faculty of Pharmacy, King Abdulaziz University, Jeddah, Saudi Arabia; ^5^ Department of Pharmaceutics, School of Pharmaceutical Education and Research, New Delhi, India

**Keywords:** breast cancer, solid lipid nanoparticles, cancer, targeted drug delivery, tamoxifen

## Abstract

Breast cancer is conventionally treated by surgery, chemotherapy and radiation therapy followed by post operational hormonal therapy. Tamoxifen citrate is a best option to treat breast cancer because its selective estrogen receptor modulation activity. Owing to its antiestrogenic action on breast as well as uterine cells, Tamoxifen citrate shows uterine toxicity. The dose 20 mg per day of Tamoxifen citrate required to show therapeutic effect causes side effects and toxicity to vital organs such as liver, kidney and uterus. In the present study, transferrin-conjugated solid lipid nanoparticles (SLNs) were successfully prepared to enhance the active targeting of tamoxifen citrate in breast cancer. Developed formulations were evaluated for particle size, surface charge, surface morphology and *in vitro* dissolution studies. Developed formulations exhibited more cytotoxicity as compared to pure Tamoxifen citrate solution in time as well as concentration dependent manner on human breast cancer MCF-7 cells. Further, cell uptake and flow cytometry studies confirmed the qualitative uptake of developed D-SLN and SMD-SLN by human breast cancer MCF-7 cells. Overall, proposed study highlights that transferrin engineered nanocarriers could enhance the therapeutic response of nanomedicines for breast cancer treatment.

## Introduction

Now a day’s advancement and expenditure in cancer research is growing year by year because changing lifestyle and many other factors causing serious life-threatening cancer. Cancer incidence progressively increasing worldwide. Breast cancer is most occurred cancer in women as compared to other different types of cancers, hence currently breast cancer is most focused area of interest for research (Devi et al., 2020). National Cancer Registry Program in India estimated maximum number of patients are in the younger age groups i.e. in their thirties and forties. As per WHO report breast cancer has second highest reported cases incident among all cancer cases. Approximately 627,000 women death was reported due to breast cancer in 2018, which is about 15% of total death among women. Frequency of breast cancer cases is comparatively high in developed region of the world which slowly spreading to neighboring countries. In US one among eight women will be patient of breast cancer in course of her life period. Fatality rate of breast cancer is high to US women as compare to other cancer, beside lung cancer (Tagde et al., 2020). The maximum mortality is claimed in Estrogen Receptor Positive (ER+) breast cancer where the risk factors involved are related to age and estrogen exposure (Osborne, 1998).

When Estrogen bind to the estrogen receptor it stimulated the normal growth and division of breast tissue cells. Hormonal therapy is the first line of treatment for ER+ breast cancers and Tamoxifen citrate has been the drug of choice for four decades. Tamoxifen citrate belongs to the class of selective estrogen receptor modulators. These selectively bind to the estrogen receptors inhibit the estrogen dependent growth of breast epithelial cells and breast cancer cells. However, the challenge is to avoid estrogenic effect on other estrogen producing tissues such as the uterus leading to side effects like endometrial thickening and endometrial cancer. The other side effects of Tamoxifen citrate include pulmonary embolus, deep vein thromboses and cataract formation (Zhang et al., 2015). Tamoxifen citrate belongs to a BCS class II drug showing poor aqueous solubility and thus less bioavailability (range 15–40 ng/ml) when administered as a single oral dose of 20 mg/day (Sharma et al., 2020). The prolonged use of high dose of Tamoxifen citrate results in toxicity and accumulation in highly distributed organs such as the liver and kidneys (Howell et al., 2004).

Significant amount of research has been published to improve the bioavailability of Tamoxifen citrate by addition of cyclodextrin or solubility enhancers (Allan et al., 1985). Tamoxifen citrate loaded ternary solid dispersion was prepared using PEG 6000 and Methyl cellulose to enhance its bioavailability and solubility (Shaker, 2014). Through this type of formulations, not only the solubility of Tamoxifen citrate is enhanced but the side effect is also diminished. Advances in breast cancer therapy have led to better management of the disease with respect to detection and treatment. This in turn, has resulted in improved survival rates of the patients yet the reachability in effective concentrations at the tumor site has remained a challenge (Shin et al., 2006). The tumor shows leaky vasculature leading to accumulation of nano sized particles at the tumor site by the EPR effect (Lumachi et al., 2013; Kesharwani and Iyer, 2015). This kind of accumulation called ‘‘passive targeting’’ is only related to the particle size and is advantageous in treating cancer (Kesharwani et al., 2014; Kesharwani et al., 2015).

Various approaches such as polysaccharides, polymeric NPs, gold NPs, lipid-based NPs etc. have been tried to load Tamoxifen citrate (Jain et al., 2015; Jain et al., 2019). Neralakere et al. successfully prepared Tamoxifen citrate loaded chitosan NPs for oral administration. These NPs exhibited desired nano size and controlled biphasic release of Tamoxifen citrate (Ravikumara and Madhusudhan, 2011). Various combinations of chitosan and alginate were used to formulate Tamoxifen citrate loaded NPs. The controlled release of Tamoxifen citrate from the NPs was dependent upon concentrations of alginate and chitosan used (Martínez et al., 2013). In another attempt Dreadon and coworkers showed successful formulation of pegylated thiol gold NPs of Tamoxifen citrate. Increased in activity of Tamoxifen citrate (TC) was observed might be due to enhancements in cellular transport by endocytosis and not due to passive diffusion of free Tamoxifen citrate (Dreaden et al., 2009). Tamoxifen citrate was also formulated as topical liposome using phosphotidyl choline and cholesterol to avoid side effects after oral administration by Bhatia et al. The drug molecule has been successfully entrapped in the liposomes with reasonable loading and desired liposome vesicle characters. Jain et al. successfully developed Poly lactic glycolic acid NPs for oral administration of tamoxifen citrate. It showed 3–4 times improved bioavailability and reduced toxicity in the liver than the Tamoxifen citrate solution (Jain et al., 2011).

The present work comprised of successful development of SLN as it has attracted great attention, among the other above-mentioned colloidal drug delivery systems. SLN offer many advantages such as low toxicity (Ghasemiyeh and Mohammadi-Samani, 2018), ease of scale-up, stability and ability to entrap both, hydrophobic and hydrophilic drug candidates. SLN also give a lot of flexibility with respect to production procedure which can be modified as per the expected loading and release (Müller et al., 2000). Lipid NPs can enhance the membrane stability and also reduced the problem of drug-leaching associated with conventional emulsions and liposomes formulations (Mehnert and Mäder, 2001; Bhatia and Bhatia, 2016). Further, biodegradation and toxicity problems of polymeric NPs is also improved (Harivardhan Reddy and Murthy, 2005) thus facilitate prolonged drug release (Zur Mühlen et al., 1998). Lipid NPs are prepared from biocompatible lipids display excellent biodegradability and low toxicity. For the poorly aqueous soluble drug SLN are good carriers (Torchilin, 2010) so that lipid based nanocarrier have been selected for the delivery of hydrophobic drugs. Due to high cholesterol need of tumor cells, they showed enhanced uptake of low-density lipoproteins, which proved the importance of SLN as nanocarrier in cancer chemotherapy (Becker et al., 2015). For the preparation of SLNs HPH are mostly used, reliable and powerful technique.

The passive targeting would direct SLN to cancer cells as well as other highly active cells, thus active targeting is essential to avoid damage to healthy cells. It is very important to deliver anticancer drug to the targeted site, without distribution to the non-cancerous cells (Strickley, 2004). Further, such types of formulation may reduce the undesirable and very harmful effect of drugs. Targeted NPs were developed by conjugation with targeting ligands, i.e., folic acid, transferrin etc. is widely explored concept (Dreaden et al., 2009).

In this study we have design and develop surface modified solid lipid NPs to improve target specificity of the developed SLN. Transferrin, a glycoprotein present in blood, is attached by active conjugation to the lipid. The prepared SLN and surface modified SLN have been characterized for morphological and physicochemical parameters. The goal of this study is to combine the advantages of the drug Tamoxifen citrate and colloidal nanocarriers system viz. SLN to target the cancer cells and protect the normal cells. SLNs were prepared by HPH using physiologically acceptable and GRAS listed lipids, surfactants and stabilizers. The hypothesis was to develop a nanocarrier having not only the solid matrix but also having liquid domains combining the benefit of solid matrix (prevents drug leakage) as well as of the liquid regions (show better solubility for lipophilic drugs). The triglycerides were used at their melting range temperatures along with solubilizers to improve entrapment of drug in SLN. The surface active agent are able to solubilize drug molecules by the mechanism of either a direct cosolvent or by uptake into micelles (Mulik et al., 2010).

The *in-vitro* cytotoxic effect was assessed on MCF-7 breast cancer cell lines as these are estrogen sensitive breast cancer cell lines. The minimum inhibitory concentration values of SMD-SLN and D-SLN are compared to TC solution. MTT assay results was confirmed by other assay called wound scratch assay was also performed. Cell uptake and flow cytometry studies were done to confirm uptake of D-SLN and SMD-SLN by cells. Tamoxifen citrate has been traditionally used as oral hormonal therapy for the breast cancer treatment, Haemolytic activity of the developed formulations was assessed to confirm suitability for Intravenous route. Acute Toxicity and repeat dose toxicity studies were performed as per OECD guidelines using female Wistar rats to evaluate toxicity of the developed formulations *vis a vis* plain drug solution.

## Materials and Methods

### Materials

TC was acquired as a gift sample from Neon Laboratories Ltd. India. Precirol^®^ 5 ATO (Glyceryl Palmitostearate,gift sample from Gattefosse, India.), Cremophor EL (Gift sample from BASF India Pvt. Ltd.) were also used in the study. Transferrin was purchased from M.P. Biologicals, India. The other material of analytical grade was used and purchased from S.D. Fine Chemicals, India.

MCF-7 Breast cancer cell line was made in Advanced Cancer Research Institute, India. Chemicals required for maintaining and sub-culturing cell lines, i.e., IMDM, Fetal Bovine Serum, were procured from Genetix, India. 100 units/mL Antibiotics (Penicillin and Streptomycin) and saline-EDTA solution were procured from Genetix, Mumbai, India. Female Wistar rats required for toxicity studies were procured from Haffkine Institute Ltd. India.

### Preparation of Drug Loaded Solid Lipid Nanoparticles (D-SLN)

For the preparation of D-SLNs hot emulsification method was used. The selected BCS class II drug Tamoxifen citrate was incorporated into the lipid phase along with surfactant (1% w/v) at 10°C above the melting range of the selected lipid. The aqueous phase consisted of 0.5% w/v surfactant solution in double distilled water. Temperature of aqueous phase was kept same as lipid phase temperature. Both the phases were mixed under continuous stirring at 600–800 RPM using Magnetic stirrer (Bio Lab BL223-B) upto 1 h. To achieve further reduction and uniformity in particle size, the D-SLN were homogenized for 3cycles per minute by HPH (APV 2000 Model, Lab homogenizer), using pressure up to 400 bar.

### Preparation of Surface Modified Drug Loaded SLN (SMD-SLN), Tamoxifen Citrate Solution (TC Solution), and Blank SLNs

**FIGURE 1:**
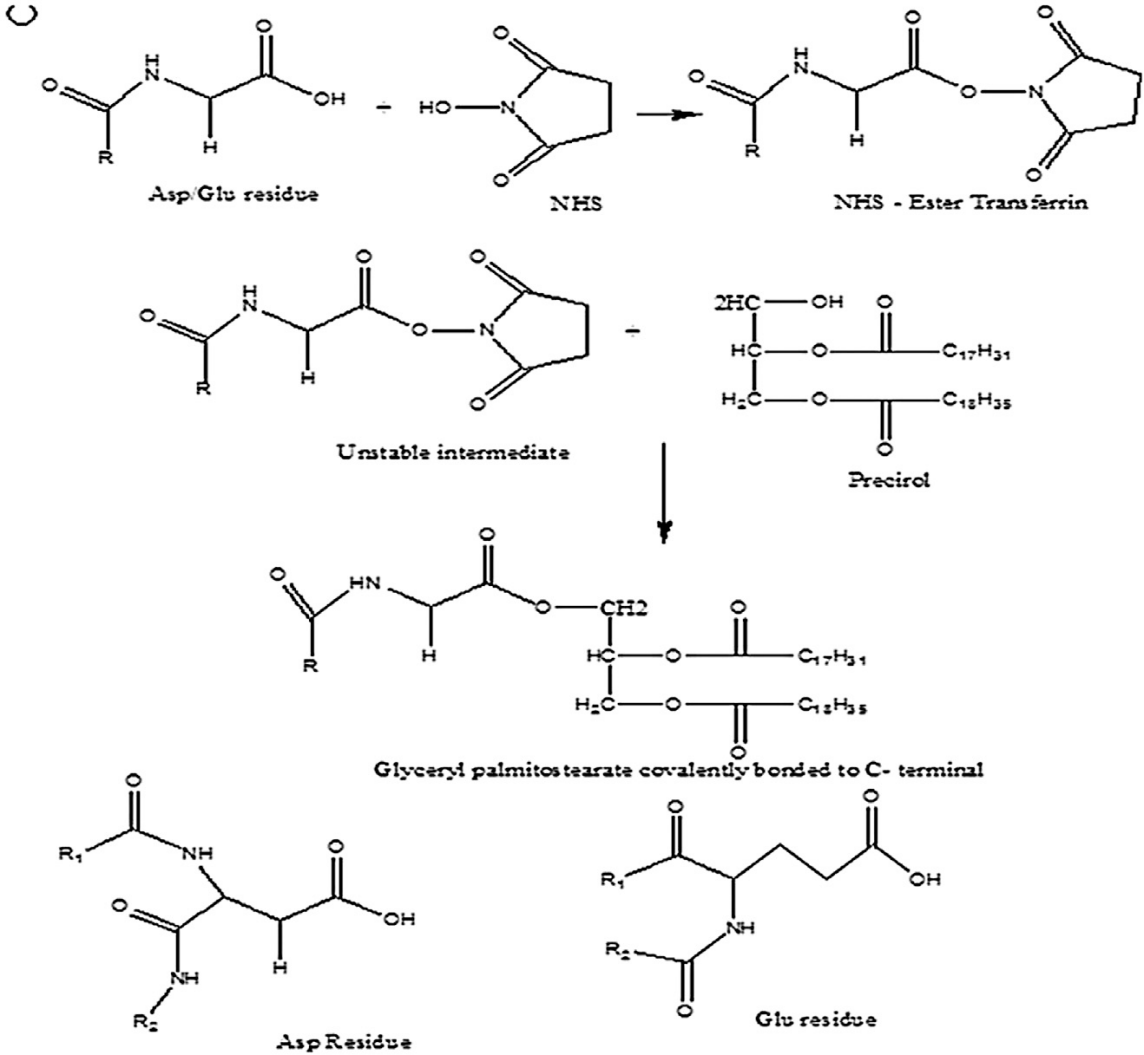
Conjugation of transferrin to lipid by covalent bonding.

The ligand selected for surface attachment is a glycoprotein, transferrin which was activated using a suitable coupling agent, DCC-NHS (Nerkar et al., 2012) and incubated with already developed D-SLN at RT for 4 h. The unattached ligand protein was removed by centrifugation at 2,000 rpm for 20 min, using Laboratory Centrifuge (Remi R-4C). This surface modified SLN will be referred as SMD-SLN (Figure 1).

The formulation was optimized by using 3^2^ factorial design. This technique is commonly used for the development of nanoparticles as a systematic approach of formulation development (Radaic et al., 2015), yet selection of variables plays critical role in designing experiments.The response surface plots were plotted using the Design Expert^®^ Version 8.0.7.1 The critical attributes were checked and particle size of the solid lipid nanoparticles was selected as the response variable. The transferrin protein attached to drug loaded nanoparticle encounters a transferrin receptor on the cell on the surface, it gets bound and transported into the cell in a vesicle by receptor mediated endocytosis. As the particle size will determine and affect the uptake of the developed nanoparticles by cancer cells. The increase in uptake is directly proportional to the anticancer activity.

Tamoxifen citrate solution is prepared by dissolving same amount of Tamoxifen citrate (1,000 µg/mL) as D-SLN and SMD-SLN in distilled water with required quantity of solubilizer. This solution will be referred as TC Solution and used for comparative studies.

Solid lipid particles were prepared, like D-SLN without addition of drug and are termed as Blank SLN to assess if any cytotoxic effect of excipients on MCF-7 cell lines.

### Sterilization and Lyophilization of SLNs

ϒ sterilization is a the most suitable and safe terminal sterilization method for sterilization of developed D-SLN and SMD-SLN as compared to other methods such as heat sterilization, sterilization by filtration etc. The sterilization was done in ϒ sterilization chamber by exposing the material to gamma rays originating from radioisotopes cobalt 60. Only one parameter i.e. time of exposure is to be controlled. A dose of 5 kGy of radiation was enough for complete sterilization (BRIT, BARC, India).

The homogenized batches of D-SLN and SMD-SLN were lyophilized using the Epsilon 2-4 LSC Lyophilizer (Martin Christ) for 32 h using 2, 3, and 4%w/v concentrations of trehalose as cryoprotectant. The batches were frozen upto −50°C for 8 h, followed by primary drying upto −20°C for 8 h and secondary drying for 16 h. These batches were reconstituted in phosphate buffer saline 7.4 and characterized for parameters like size, PDI, zeta potential, drug content, *in-vitro* drug release.

### Characterization of D-SLN and SMD-SLN

#### Particle Size Analysis and Polydispersity Index Measurements

Particle size and polydispersity index of the developed NPs were determined by using a Zetasizer [Malvern model Zen3690 (Ver 6.12)]. The average size acquired by the D-SLN and SMD-SLN, when dispersed in water is indicated by particle size whereas the homogeneity in distribution of these particles is depicted by polydispersity index. 20 μL of the D-SLN and SMD-SLN were suitably diluted with 4 mL double distilled water. The intensity of the scattered light was measured at 90° to determine hydrodynamic diameter and the PDI (Dua and Gude, 2006).

#### Zeta Potential Measurements

The surface charge in D-SLN and SMD-SLN was determine by measuring zeta potential. Malvern Zetasizer 90 S was used to determine zeta potential. The samples diluted as per the procedure discussed in particle size measurement, were taken in the quartz cuvette. Approximately 15 runs were measured at 90°.

#### Transmission Electron Microscopy (TEM)

Surface morphology and size of D-SLN and SMD-SLN were confirmed under Transmission electron microscope (Model: CM 200 Make Philips). D-SLN and SMD-SLN were stained with 1.0% w/v Phosphotungstic acid solution for better resolution. With the help of filter paper excess of reagent was drained and allowed to dry for 3–5 min at RT. The slide was placed on the TEM and was scanned for the image.

#### X-ray Diffraction (XRD) Analysis of NPs

X-ray diffractometer made of PW 1729, Philips, Netherlands was used to determine crystalline and polymorphic form of the drug. The sample of prepared formulation were examined after vacuum freeze drying (Jenning et al., 2000) at the scanning rate 2–5°C/min.

#### Drug Content and Entrapment Efficiency

Drug content of the D-SLN was analyzed by using developed analytical method. One mL of D-SLN and SMD-SLN were transferred to a stoppered test tube followed by addition of 5 mL of methanol. The supernatant obtained after centrifugation was determined for drug content by UV Spectrophotometer at 257 nm. The centrifugation of diluted sample was done at high speed of 20,000 rpm for 1 h in refrigerated centrifuge. The separated supernatant was quantified for the free drug and entrapment efficiency was obtained.

#### *In vitro* Drug Release Studies

Dialysis bag method was adapted to find quantitative data of drug release from the D-SLN and SMD-SLN and, the complete release profile of D-SLN (n = 6). Weighed amount of the D-SLN, SMD-SLN and TC solution were transferred to a dialysis bag L-(Mol. Wt cutoff 6,000–8,000 Dalton, Hi media), sealed and suspended in a beaker having dissolution medium (pH 7.4 Phosphate Buffer) under continuous stirring at 100 rpm maintained at a temperature 37°C ± 2°C by the heater. Aliquots were taken at predetermined intervals and same amount was replaced with fresh medium. The percent cumulative drug release was analyzed at each time point for 120 h, using predeveloped UV spectrophotometric method at 257 nm. Graphs indicating the % drug release vs time were plotted. The *in-vitro* release profiles of both the D-SLN and SMD-SLN were compared with that of the TC Solution.

### Cell Culture

Cell culture is maintained at 37°C in humidified atmosphere of 5% CO_2_ in IMDM supplemented with 10% Fetal Bovine Serum. Added antibiotics to the cell culture medium. Saline-EDTA solution was used to sub-culturing of cells after removing from flask.

#### MTT Cytotoxicity Assay

MTT assay was used to evaluate the cytotoxicity of drug as previously reported (Franken et al., 2006; Goel and Gude, 2011). MCF -7 breast cancer cells/well were seeded in a 96-well plate after 24 h cells were treated with TC solution, blank SLN, D-SLN and SMD-SLN at varying doses of 50–0.01 μg/mL. Plates were incubated at 37°C for 24, 48 and 72 h in 5% CO_2_ incubator. Formulations were aspirated, and the wells were washed twice with PBS at respective time points followed by treatment with MTT as per reported methods. The optical density was measured, IC_50_, was calculated and results was interpreted.

#### Wound Scratch Assay

It was performed to check proliferation of cells (Franken et al., 2006; Mulik et al., 2012; Jain et al., 2013). Around, 0.6 million cells were seeded in 35 mm plates. Further, next day cells were treated with 2 μg/mL mitomycin C for 1 h. The cells in the center of the plates were later scraped by sterile tip to form a wound. Sub-toxic doses of TC solution (μg/mL), D-SLN and SMD-SLN were added to the plates and plate were incubated for 24 h. The width of the wound was measured by using Axio Vision Rel 4.8 imaging software. The obtained result was plotted as percent wound closure compared to control. The controls were untreated samples and considered to be covered 100%. And the results were compared with control samples.

#### Confocal Microscopy

Sub-confluent cultures of MCF –7 cells were grownup on coverslips and treated with D-SLN and SMD-SLN (FITC loaded). Cells were washed twice with PBS and fixed with 1% w/v paraformaldehyde. Washing was done with PBS thrice and coverslips were later mounted using 2.5% w/v DABCO on glass slides and sealed using nail paint. Acquisition was done on confocal microscope (LSM 510) and LSM image browser software was used for data analysis.

### Cellular Uptake Using Flow Cytometry

Sub-confluent cultures of MCF seven cells on plates were treated with formulations (FITC loaded) for 30 and 60 min respectively. Cells were harvested at specific time points and fixed using 1% PFA at 37 °C for 15 min. Cells were washed twice using PBS and finally suspended in the same. Acquisition was done using FACS and results were analyzed by Cell Quest software. Cellular uptake and cell cycle revealed the exact stage at which the cell growth is arrested.

### Stability Studies

The nanoparticles were kept for stability studies as lyophilised powder and the stability studies were conducted on reconstituted lyophilised formulation. The medium used for reconstitution is phosphate buffer saline pH 7.4 The protocol for stability studies was as per the ICH Q1A R2 guidelines for stability studies of new drug substances and products; for drug products intended to be stored under refrigeration. The samples were kept for long term studies at refrigeration (5°C ± 3°C) and for accelerated studies at 25°C ± 2°C/60% ± 5% RH. The samples were withdrawn at the time point 0, 1, 2, 3, and 6 months and evaluated for various physicochemical parameters such as particle size, zeta potential, drug content, entrapment efficiency and drug release.

## Results and Discussion

### Selection of Excipients

Drug loaded SLNs (D-SLN) and Tamoxifen citrate loaded surface modified SLNs (SMD-SLN) were successfully formulated and anticancer activity of the same is assessed against pure drug solution. Various glycolipids such as glyceryl behenate, stearic acid was screened and glyceryl palmitostearate, an FDA approved diacyl glyceride is shortlisted as it yielded SLN with best physicochemical properties. This glyceryl palmitostearate is present in four crystalline structures with most thermodynamically stable β-form. Polyoxyl 35 castor oil (Ethoxylated Castor oil) is a nonionic solubilizer that aids solubilization of Tamoxifen citrate in the selected lipid. It is also an emulsifying agent with HLB value 12–14 and acts as hydrophobic emulsifier in current lipid based system. To balance the HLB value of lipid based system, water soluble surfactant such as polysorbate 80 with HLB value 15 and IIG limit 10% is added. Addition of liquid or semisolid emulsifier such as polysorbate 80 were reported to hasten transformation from *α* to *β* form owing to increased molecular mobility.

### Preparation of SLNs Formulation

Plain SLN were successfully prepared by hot emulsification method. Batch optimization was done based the results of PSD, zeta potential and PDI of all batches. From here optimized batch was used for the further study.

### Preparation of Surface Modified Drug Loaded SLN (SMD-SLN by Tf Conjugation)

Tf was engineered on the periphery of SLNs by reaction using coupling agent. Tf was first mixed with N,N′-Dicyclohexylcarbodiimide (DCC) and N-hydroxysuccinimide (NHS), in exact proportions in dichloromethane. At this step, COOH group of Tf reacts with DCC to form an amine reactive O-acylisourea intermediate. This intermediate then reacts with O–H group of Glyceryl behenate on the surface of SLNs. Stable ester bond between Tf and SLNs was formed when the Tf-DCC mixture was added to the SLN suspension. by-product is released as a soluble urea derivative (Reid, 1998) (Figure 1). Conjugation of Tf to lipids was confirmed by three different methods, i.e., Bradford assay, NMR spectrum and IR spectrum (data not included).

The selection of lipid was done based on the solubility of drug in lipid, but the concentration of lipid used is important for entrapment efficiency of the drug in the lipid. The concentration of surfactant determines the encapsulation as well as stability of drug in the nanoparticle. Thus, the lipid concentration (1–1.5% w/v) and surfactant concentration (0.15–0.5% w/v) were considered as formulation variables whereas the particle size of the dispersion was considered as response variable. The drug concentration was kept constant at 0.1% w/v for all the experiments. The selection of optimized batch was based on the particle size (Supplementary Table S1).

### Physicochemical Characterization

#### Particle Size, Polydispersity Index, Zeta Potential and TEM Images

**FIGURE 2:**
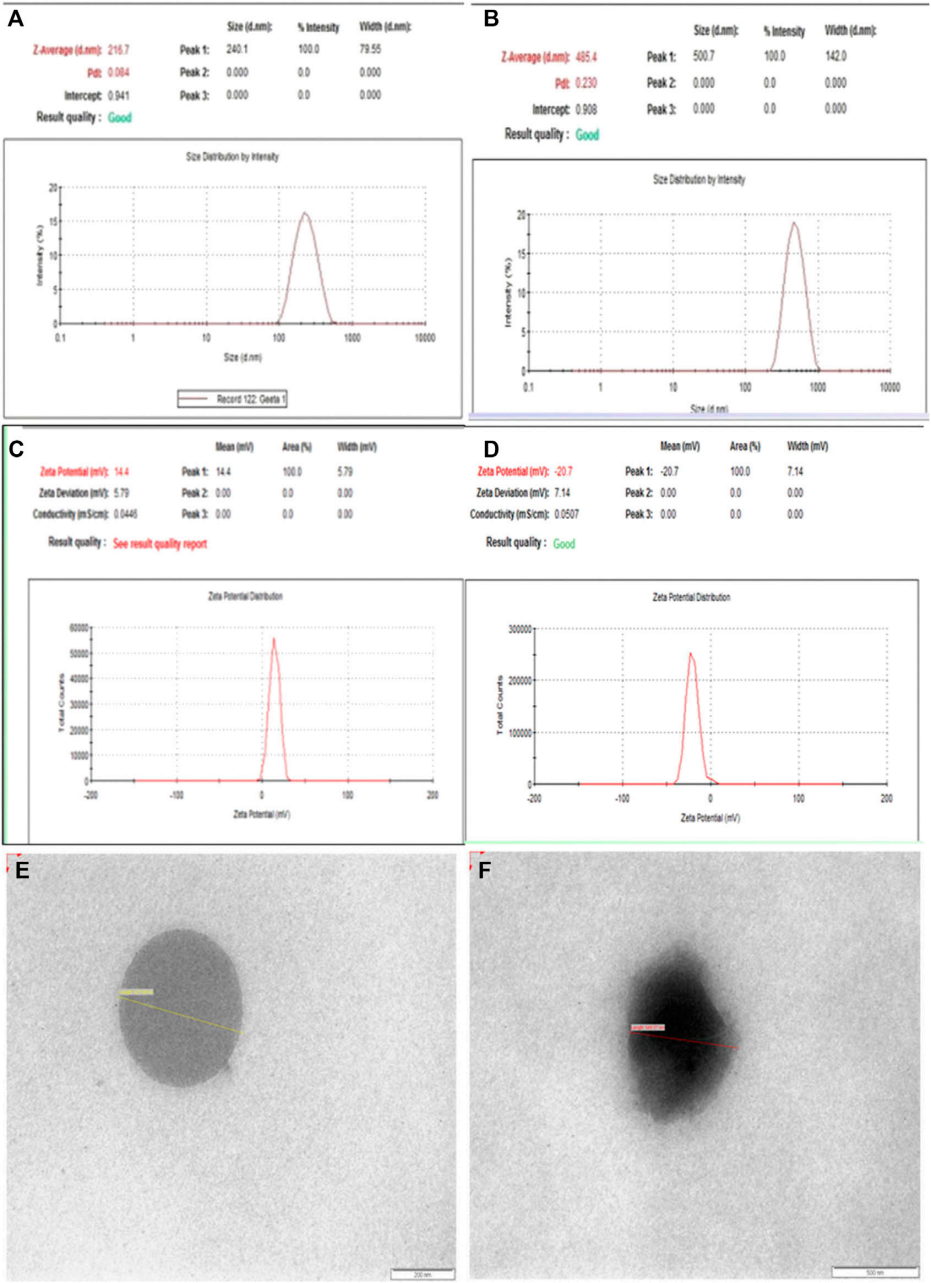
Size **(A,B)**, zeta potential **(C,D)** and TEM images **(E,F)** of D-SLN and SMD-SLN.

The mean particle size of D-SLN prior to homogenization was 231 ± 23 nm with polydispersity index 0.220 ± 0.05 with 88.5% drug content and 73% entrapment efficiency (Figure 2A). After homogenization, the particle size and particle size distribution were decreased to 216 ± 32 nm with polydispersity index 0.048 ± 0.03. Homogenization at higher pressure increased the temperature of system and again increased the particle size of D-SLN. The surface modified solid lipid particles showed average particle size 485 ± 43 nm with polydispersity index 0.230 ± 0.05 with 69% entrapment efficiency and 85% drug content (Figure 2B).

The zeta potential was obtained −7.0 ± 0.05 mV and −20.5± 0.05 for control and Tf-C-SLN respectively (Figures 2C,D). tr was shifted toward more negative side after conjugation to the SLNs. The entrapment efficacy for the prepared SLNs was found to be 73 ± 2% that is considered as good encapsulation efficiency. The percentage Tamoxifen citrate remained unaffected after 3 and 6 months in D-SLN and SMD-SLN. 6 months stability study data revealed that there was not much change in physical parameter which prepared formulation have enough stability.

The surface morphology of SLNs, D-SLN and SMD-SLN was confirmed by TEM images (Figures 2E,F). The images were clearly seen using contrasting agent phosphotungstic acid at magnification 100,000×. Figure 2E showed the perfectly spherical shape of D-SLN, however, Figure 2F showed hazy image due to ligand attached to the surface of SMD-SLN. Zeta potential of D-SLN were and that of SMD-SLN were found to be. More negative values of zeta confirmed more stable SLN as charged particles repel each other and added to thermodynamic stability.

#### PXRD Characterization

The diffraction pattern showed majority of less ordered crystal, of tamoxifen citrate in D-SLN and SMD-SLN, due to non-crystalline, amorphous or less ordered structure formulation would increase the drug loading capacity. Tamoxifen citrate XRD diffraction was different from those of D-SLN. The sharp peaks of Tamoxifen citrate, at 9.256°, 11.418°, 13.571°, 15.968°, 17.495°, 21.071° (2*θ*) confirmed the crystalline nature of TC, whereas these sharp peaks were not showed in D-SLN and SMD-SLN, it means that Tamoxifen citrate present in amorphous form into the formulation in the form of molecular dispersion in lipid core. Diffraction pattern of B-SLN and D-SLN were not more different from the pure tamoxifen citrate. Crystalline nature of the tamoxifen citrate was changed to more soluble amorphous form as result of encapsulation into SLNs. PXRD study revealed that the drug into prepared formulation was found to be in amorphous form.

#### *In vitro* Drug Release

PBS pH 7.4 was the medium selected for *in vitro* studies as it has maintained the required sink condition for the drug Tamoxifen citrate. *In vitro* release study showed steady release in D-SLN while initial burst release for the first 1–2 h is observed in SMD-SLN which can be obtained due to the adsorption of drug into SLNs surface. Sustained drug release pattern was observed after 6 h from SLNs. The drug release was found to be 48 ± 3% and 56 ± 2% for D-SLN and SMD-SLNs respectively after 48 h (Figure 4A). There is no significant difference in the release pattern of both SLNs. In 120 h, the drug release from the TC solution is only 50% (*n* = 5), whereas D-SLN showed sustained drug release upto 95% and SMD-SLN shows 92% drug release in 120 h. The entrapment of Tamoxifen citrate shows improvement in drug release as compared to TC solution. Thus, more than 90% of drug is available for therapeutic effect.

### Cell Culture Studies

Estrogen sensitive MCF-7 breast cancer cell lines were used to conduct *in vitro* cytotoxicity studies as Tamoxifen citrate belongs to SERM and acts on estrogen receptors. Cytotoxicity of D-SLN and SMD-SLN was performed by *in vitro* cell line studies such as MTT assay, wound scratch assay and cell uptake assays.

#### Cellular Proliferation Using MTT Assay

**FIGURE 3:**
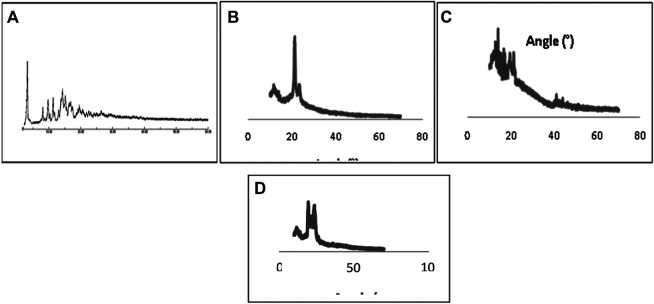
X-ray diffraction pattern of Pure drug **(A)**, Tamoxifen citrate **(B)**, B-SLN **(C)**, D-SLN and SMD-SLN **(D)**.

The MTT assay was used to assess the antiproliferative action of Tamoxifen citrate at increased concentration. To determine the IC_50_ of the TC solution, blank SLN, D-SLN and SMD-SLN for 24, 48, and 72 h respectively, the MTT studies were carried out which were dependent on time and dose. The antiproliferative activity of TC solution, D-SLN and SMD-SLN, were exhibited dose dependent. There was no significant difference in the anticancer activity was observed at the lowest dose (0.1–1.0 µg/ml), but significant variation in activity was observed at the concentrations above 5 µg/ml from these formulations. The cell viability was observed 93.3 ± 2% and 86.7 ± 2.4%, for TC solution and D-SLN respectively, at the dose of 5 µg/ml. At the dose of 9 µg/ml, the effect was comparatively more noticeable. The reduction in cell viability were 80 ± 2.2% and 48.3 ± 1.3% with TC solution and with D-SLN. D-SLN showed significance higher reduction in cell viability as compare to TC solution, might be due to formulation released tamoxifen in proper manner. The cellular viability was almost same with SMD-SLN. Difference in reduction in cell viability was significant more with D-SLN and SMD-SLNs as compare to TC solution at 20 µg/ml dose. After 24 h of treatment with SMD-SLN the cell viability was 100 ± 2%, compared to 50 ± 1.5% with D-SLN and TC solution while showing further reduced antiproliferative effect of D-SLN and SMD-SLN at 48h and at 72h, respectively due to extended release. By using different concentration of TC solution, D-SLN and SMD-SLN was studied for antiproliferative activity with respect to time. Since, there were negligible effect of controls on cell viability, but due to presence of Tamoxifen citrate in the formulations all the three formulations exhibited antiproliferative effect against the cancer cell (Figures 3B **,C,** 4B **,C**).

**FIGURE 4:**
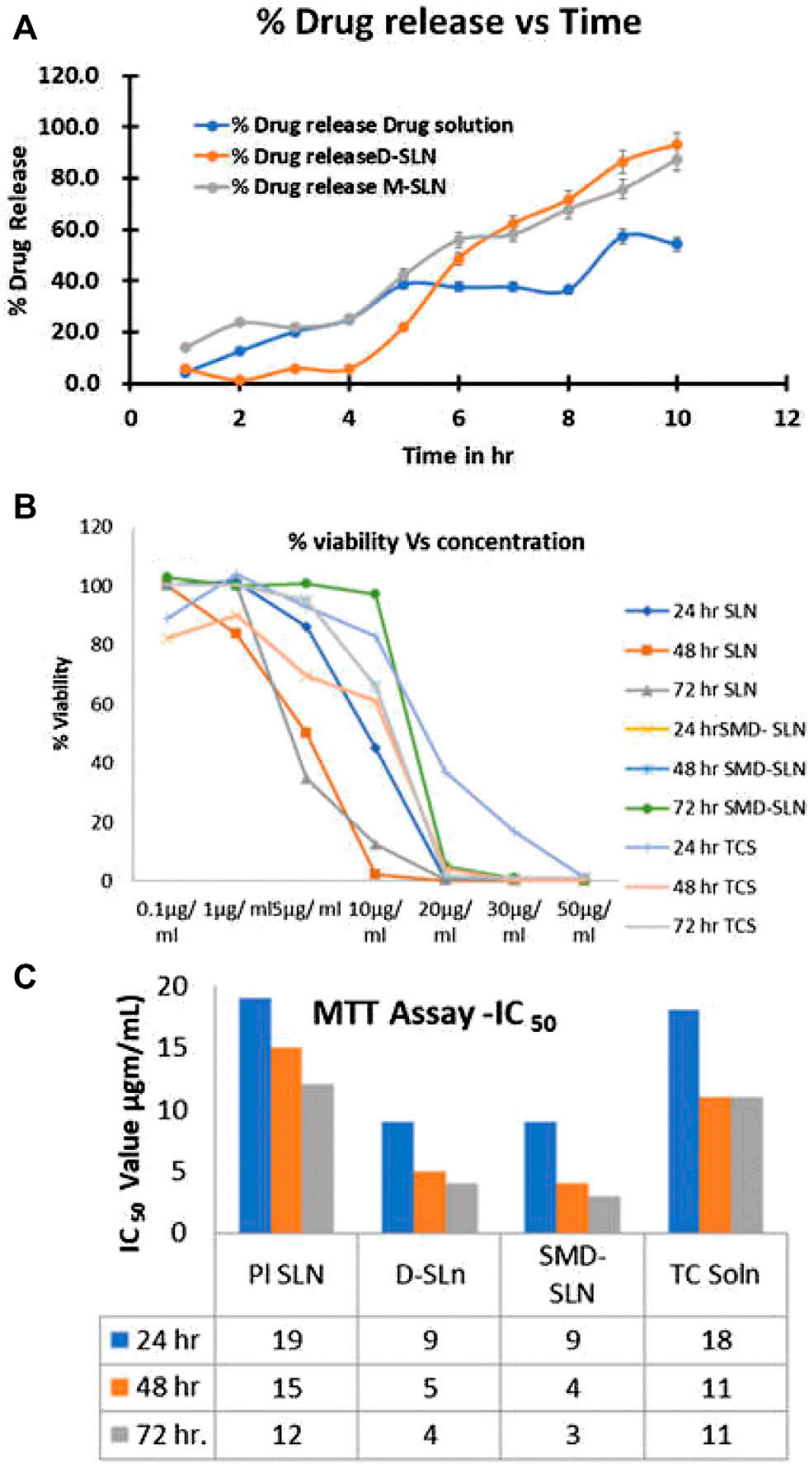
*In vitro* drug release profile **(A)**, cell viability **(B)**, and IC50 graph of developed SLN formulations.

The observed IC_50_ values were 18 μg/ml for TC solution on MCF seven breast cancer cell lines, but, when the equal drug loaded in the nanoparticles, IC_50_ values reduced to 9, 5, and 4 μg/ml at 24, 48, and 72 h for D-SLN and 9, 4, and 3 μg/ml for SMD-SLN at 24, 48, and 72 h respectively. As shown in figure, as the time of exposure increased, there was no change in IC_50_ value of TC solution, but IC_50_ value of D-SLN and SMD-SLN further decreased upto 48 h and stabilized at 72 h. *In vitro* drug release profile showed the change in the activity between the drug alone and drug into the formulation, it might be due to sustained release of drug from the formulation.

#### Wound Scratch Assay

**FIGURE 5:**
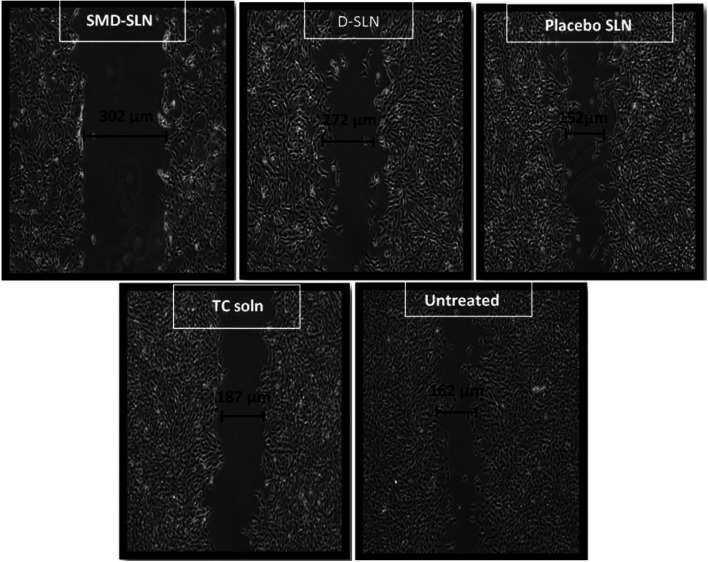
Wound coverage results of developed SLN formulations in wound scratch assay.

The formulation of D-SLN and SMD-SLN showed reduction in cell motility in wound scratch assay as compared to the untreated control (100% migration) and TC solution and blank SLN. The wound coverage in case of D-SLN, SMD-SLN, TC solution and blank SLN were found to be 48.74, 55.92, 78.74, and 85.45%, respectively. Figure 5 shows the actual cell migration after treatment with developed formulation. Cell migration of SLN without drug (B-SLN) was done to verify if any cytotoxicity was imparted by excipients added to prepare SLN. Significant (*p* < 0.05) reduction in motility of cells is seen when exposed to drug (Figure 5).

#### Cell Uptake Using Confocal Microscopy

**FIGURE 6:**
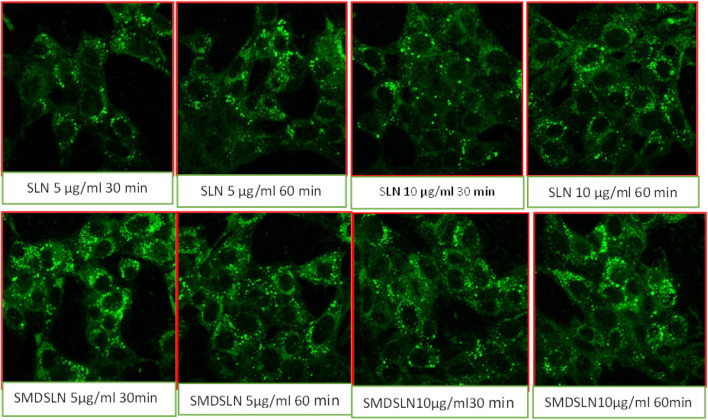
Qualitative cell uptake of developed SLN formulations by confocal microscopy.

The cellular uptake was carried for the qualitative determination of Tamoxifen citrate from D-SLN and SMD-SLN by using fluorescence microscopy and more sustained cellular uptake of TC was found from the prepared formulation (Figure 6). The intensity of fluorescence was good after 5 and 10 min, which was reduce with time, when the cell was treated with TC solution. Significant reduction in fluorescence intensity was found after one and two days (24–48 h). the enhancement in intensity of fluorescence was found after 6 h and sustained it same even after 48 h when cells treated with D-SLN and SMD-SLN, this result suggested the sustained release and sufficient retention of encapsulated TC into the targeted cells. The SMD-SLN showed higher the fluorescence intensity then D-SLN after 24 and 48 h (Data not included), it might be due to receptor mediated endocytosis of SMD-SLN. The figure clearly shows the nanoparticulate uptake by the cancer cells as green fluorescence supporting the MTT results. To see the possible autofluorescence images of untreated cells were also taken, in untreated control cells there were no autofluorescence was observed. Figure 6 clearly shows the nanoparticulate uptake by the cancer cells as green fluorescence.

#### Cell Uptake Using Flow Cytometry

**FIGURE 7:**
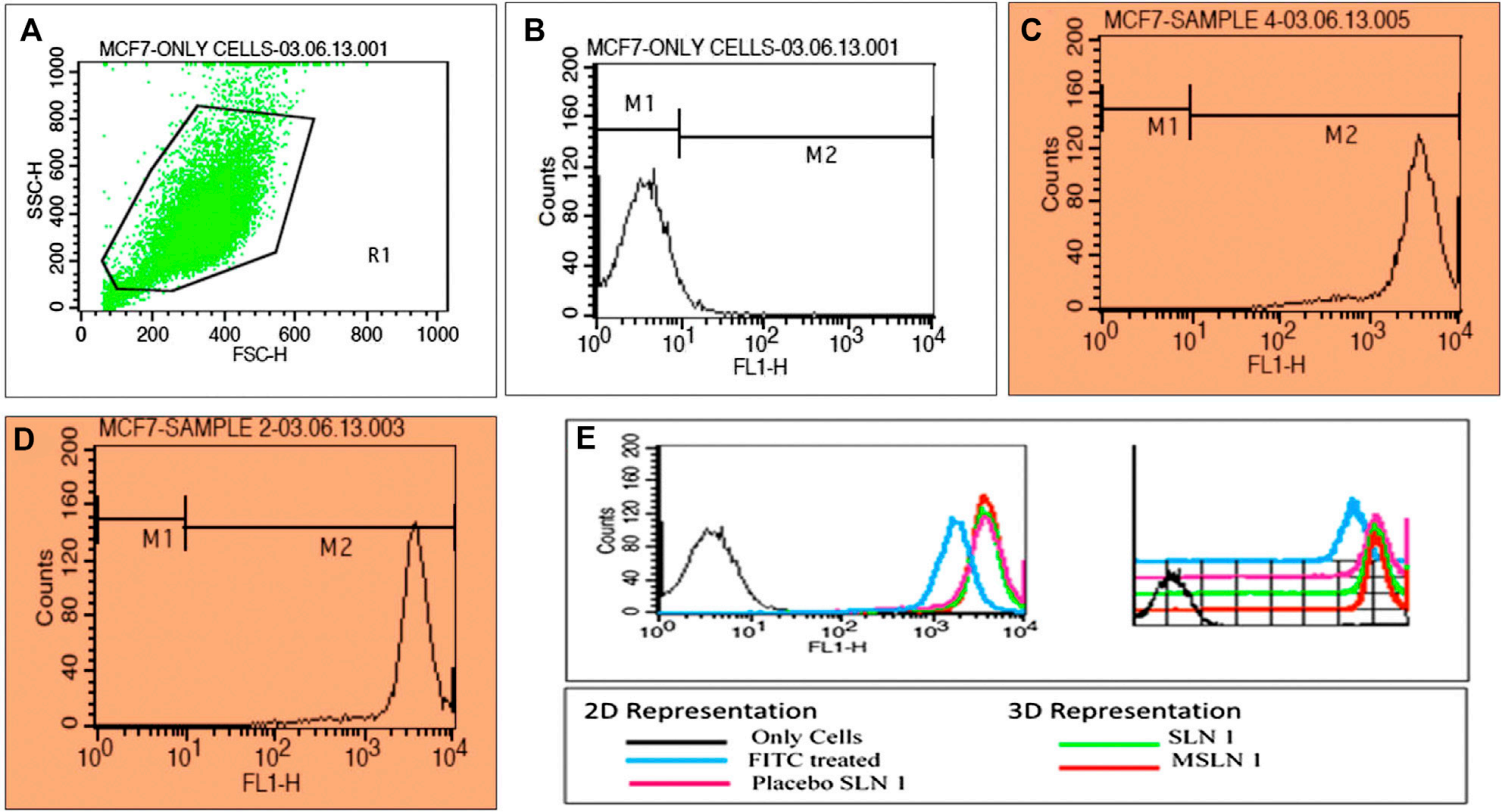
Cell uptake results of developed SLN formulations by flow cytometry. **(A)** Uptake by MCF-7 cells, **(B)** uptake by untreated MCF-7 cells, **(C)** uptake of D-SLN by MCF-7 cells, **(D)** uptake of SMD-SLN by MCF-7 cells, and **(E)** flow cytometry analysis 2D and 3D representation.

For the quantitative cellular uptake assessment of prepared nano-formulation, the flow cytometry technique was used. The mean fluorescence intensity was obtained 120, 150, and 140 units for the cell treated with FITC, with dye loaded D-SLN and with dye loaded SMD-SLN respectively. The enhancement in the intensity shows better uptake. Uptake of NPs is higher at the initial M2 level of growth cycle. In the cell cycle study, the growth is arrested at M2 level in D-SLN and SMD-MSLN (Figure 7).

Thus, increased in the intracellular uptake of tamoxifen citrate through Tf-conjugated drug delivery system is not sufficient to enhance the therapeutic benefit of the TC but retention of drug into cells in the solubilized form is also very important without this therapeutic benefit of TC would not achieved (Sahoo and Labhasetwar, 2005). The different cellular mechanism for this system might be a most probable reason for this effect. The mechanism for drug transport to the cell from transferrin-conjugated system, unconjugated system (without TF) and from simple solution were found to be receptor mediated endocytosis, nonspecific uptake mechanism and passive diffusion respectively. These different mechanisms might be responsible for change in the pharmacokinetic of the drug by these formulations. In last this study was revealed that SMD-SLN was superior comparatively to delivery of anticancer drug TC with enhanced antiproliferative activity.

### Stability Studies

The stability studies results showed that after reconstitution the particle size has increased upto 15% in D-SLN whereas it has increased by 10–15% in M-SLN. The zeta potential and pH has not changed much depicting good stability. The drug content and entrapment efficiency of the nanoparticle is reduced by 8–10% in D-SLN whereas the drug release at 120 h is not much affected at accelerated stability in both D-SLN and M-SLN at accelerated condition (25°C ± 2°C/60% ± 5% RH). At 5°C ± 3°C, the particle size shows increase but other critical parameters such as zeta potential, drug content, %entrapment efficiency and % drug release are not much affected in both D-SLN and M-SLN. This ensures good stability of the developed SLNs. The real time studies are ongoing for the developed nanoparticles upto 12 months (Supplementary Table S2).

## Conclusion

Based upon our results, we can suggest that prepared transferrin-conjugated solid lipid nanoparticles (SLNs) has significant potential to deliver the Tamoxifen citrate in the treatment of breast cancer with improved therapeutic activities. Based upon the results of our studies, we can suggest that our proposed targeted SLNs formulation is appropriate for targeted drug delivery of an anticancer drug considering the aspects of an ideal drug delivery system i.e. sustained drug release, targeting effect, biocompatible and biodegradable properties. The increased targeting affinity of Tamoxifen citrate against breast cancer cells MCF-7 confirmed the potential of our developed SLN for breast cancer therapy. In future, transferrin targeted drug delivery NPs can be used for treatment of other cancers as well.

## Data Availability

The raw data supporting the conclusion of this article will be made available by the authors, without undue reservation.
