# Corrigendum: Formulation and development of transferrin targeted solid lipid nanoparticles for breast cancer therapy

**DOI:** 10.3389/fphar.2022.1123542

**Published:** 2023-01-11

**Authors:** Geeta S. Bhagwat, Rajani B. Athawale, Rajeev P. Gude, Shadab Md, Nabil A. Alhakamy, Usama A. Fahmy, Prashant Kesharwani

**Affiliations:** ^1^ H. K. College of Pharmacy, Mumbai, India; ^2^ Prin. K. M. Kundanani College of Pharmacy, Mumbai, India; ^3^ Advanced Centre for Treatment Research and Education in Cancer, Tata Memorial Centre, Navi Mumbai, India; ^4^ Department of Pharmaceutics, Faculty of Pharmacy, King Abdulaziz University, Jeddah, Saudi Arabia; ^5^ Department of Pharmaceutics, School of Pharmaceutical Education and Research, Jamia Hamdard, New Delhi, India

**Keywords:** breast cancer, cancer, solid lipid nanoparticles, tamoxifen, targeted drug delivery

In the published article, there was an error in Figure 1 as published. The figure erroneously contained two duplicate images**.** The corrected Figure 1 and its caption appear below.

**FIGURE 1:**
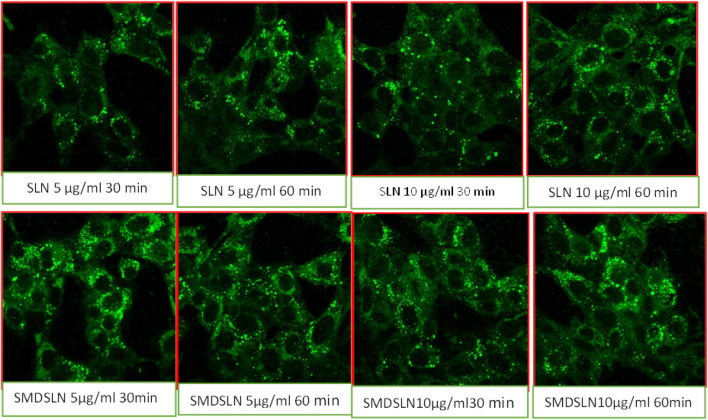
Qualitative cell uptake of developed SLN formulations by confocal microscopy.

The authors apologize for this error and state that this does not change the scientific conclusions of the article in any way. The original article has been updated.

