# Supplementary material for: Formulation and Development of Transferrin Targeted Solid Lipid Nanoparticles for Breast Cancer Therapy
Source: Front Pharmacol. 2020 Nov 27;11:614290. doi: 10.3389/fphar.2020.614290 (PMC7729133; doi:10.3389/fphar.2020.614290)
Supplement: Supplementary file 1 [file Table1.DOCX]

**Supplementary file:**

**Table S1: Selection of optimized batch**

| **S. No.** | **Cremophor EL (%w/v)** | **Lipid (%w/v)** | **Particle size (nm)** |
| --- | --- | --- | --- |
| 1 | 0 | -1 | 750 |
| 2 | 0 | 0 | 1000 |
| 3 | -1 | -1 | 1010 |
| 4 | -1 | 1 | 1200 |
| 5 | 1 | 1 | 1450 |
| 6 | -1 | 0 | 1150 |
| 7 | 0 | 1 | 1340 |
| 8 | 1 | 0 | 650 |
| 9 | 1 | -1 | 500 |

**Table S2: Stability Study for Developed SLN formulations**

| **(a) Stability Study Result for Batch D-SLN at 5°C±3°C** | | | | | | | |
| --- | --- | --- | --- | --- | --- | --- | --- |
| **Parameters** | **Initial** | **1 Month** | | | **2 Month** | **3 Month** | **6 Month** |
| **Appearance** | Clear translucent | Clear translucent | | | Clear translucent | Clear translucent | Clear translucent |
| **pH** | 6-7 | 6-7 | | | 6-7 | 6-7 | 6-7 |
| **Average particle size** | 215±32 nm | 220±34 nm | | | 227±24nm | 229±16 nm | 232±24 nm |
| **PDI** | 0.220±0.07 | 0.224±0.08 | | | 0.230±0.06 | 0.234±0.08 | 0.237±0.1 |
| **Zeta potential** | -14.0±3.3 | -14.0±3.2 | | | -15.0±4.3 | -15.0±2.3 | -15.0±3.5 |
| **Drug content** | 88.0± 2% | 87.4± 2% | | | 87.1± 2% | 86.7± 2% | 86.5± 2% |
| **Entrapment**  **efficiency%** | 73.0± 5% | 72.4± 4% | | | 72.1±3% | 71.5± 5% | 71.2± 4% |
| **Drug release** | 92.5± 5% | 92.8± 5% | | | 92.6± 5% | 92.5± 5% | 92.5± 5% |
| **(b) Stability Study Result for D-SLN at 25°C±2°C / 60%±5% RH** | | | | | | | |
| **Parameters** | **Initial** | | | **1 Month** | **2 Month** | **3 Month** | **6 Month** |
| **Appearance** | Clear translucent | | | Clear translucent | Clear translucent | Clear translucent | Clear translucent |
| **pH** | 6-7 | | | 6-7 | 6-7 | 6-7 | 6-7 |
| **Particle size** | 215±32 nm | | | 217±14 nm | 223±24nm | 229±16 nm | 232±24 nm |
| **PDI** | 0.1±0.05 | | | 0.1±0.04 | 0.1±06 | 0.1±0.05 | 0.1±0.07 |
| **Zeta Potential** | -14.0±3.6 | | | -14.0±4.2 | -15.0±5 | -16.0±5 | -16.0±5 |
| **Drug content (%)** | 94.0± 2% | | | 92.0± 2% | 90.0± 2% | 87.0± 2% | 85.0± 2% |
| **Entrapment**  **Efficiency (%)** | 73.0± 5% | | | 72.5± 5% | 72.2± 4% | 71.5± 5% | 71.2± 5% |
| **Drug release** | 92.5± 5% | | | 93.5± 5% | 91.5± 5% | 92.5± 5% | 90.5± 5% |
| **(c) Stability Study Result for Batch M-SLN at 5°C±3°C** | | | | | | | |
| **Parameters** | **Initial** | | | **1 Month** | **2 Month** | **3 Month** | **6 Month** |
| **Appearance** | Clear translucent | | | Clear translucent | Clear translucent | Clear translucent | Clear translucent |
| **pH** | 6-7 | | | 6-7 | 6-7 | 6-7 | 6-7 |
| **Average particle size** | 485±46 nm | | | 492±26nm | 495±25 nm | 502±16 nm | 506±43 nm |
| **PDI** | 0.230±0.05 | | | 0.235±0.05 | 0.241±0.04 | 0.245±0.05 | 0.248±0.07 |
| **Zeta potential** | -20.0±3.3 | | | -21.0±3.2 | -21.4±4.3 | -21.7±2.3 | -21.7±3.5 |
| **Drug content** | 85.0± 2% | | | 84.4± 2% | 84.1± 2% | 83.8± 2% | 83.5± 2% |
| **Entrapment**  **efficiency (%)** | 69.0± 4% | | | 68.5± 3% | 68.3± 4% | 68.0± 5% | 67.5± 4% |
| **Drug release** | 93.5± 5% | | | 92.8± 5% | 92.6± 5% | 92.5± 5% | 92.5± 5% |
| **(d) Stability Study Result for M-SLN at 25°C±2°C / 60%±5% RH** | | | | | | | |
| **Parameters** | **Initial** | | **1 Month** | | **2 Month** | **3 Month** | **6 Month** |
| **Appearance** | Clear translucent | | Clear translucent | | Clear translucent | Clear translucent | Clear translucent |
| **pH** | 6-7 | | 6-7 | | 6-7 | 6-7 | 6-7 |
| **Average Particle size** | 390±46 nm | | 412±37 nm | | 415±25nm | 425±16 nm | 430±33 nm |
| **PDI** | 0.1±0.05 | | 0.1±0.02 | | 0.1±0.07 | 0.2±0.03 | 0.1±0.03 |
| **Zeta Potential** | -20.0±3.3 | | -20.0±3.2 | | -20.0±4.3 | -21.0±2.3 | -21.0±3.5 |
| **Drug content** | 85.0± 3% | | 84.8± 2% | | 84.3± 3% | 84.0± 2% | 83.5± 2% |
| **Entrapment**  **Efficiency%** | 69.0± 4% | | 69.05± 3% | | 68.3± 4% | 68.0± 5% | 67.5± 4% |
| **Drug release** | 93.5± 5% | | 92.8± 5% | | 92.6± 5% | 92.5± 5% | 92.5± 5% |
